# Challenging Disease Ontology by Instances of Atypical *PKHD1* and *PKD1* Genetics

**DOI:** 10.3389/fgene.2021.682565

**Published:** 2021-06-25

**Authors:** Jonathan de Fallois, Ria Schönauer, Johannes Münch, Mato Nagel, Bernt Popp, Jan Halbritter

**Affiliations:** ^1^Department of Endocrinology, Nephrology and Rheumatology, University of Leipzig Medical Center, Leipzig, Germany; ^2^Center for Nephrology and Metabolic Disorders, Weißwasser, Germany; ^3^Institute of Human Genetics, University of Leipzig Medical Center, Leipzig, Germany

**Keywords:** PKD1, PKHD1, ADPKD, ARPKD, cystic kidneys, chronic kidney disease

## Abstract

**Background:**

Autosomal polycystic kidney disease is distinguished into dominant (ADPKD) and recessive (ARPKD) inheritance usually caused by either monoallelic (*PKD1*/*PKD2*) or biallelic (*PKHD1*) germline variation. Clinical presentations are genotype-dependent ranging from fetal demise to mild chronic kidney disease (CKD) in adults. Additionally, exemptions from dominant and recessive inheritance have been reported in both disorders resulting in respective phenocopies. Here, we comparatively report three young adults with microcystic-hyperechogenic kidney morphology based on unexpected genetic alterations beyond typical inheritance.

**Methods:**

Next-generation sequencing (NGS)-based gene panel analysis and multiplex ligation-dependent probe amplification (MLPA) of PKD-associated genes, familial segregation analysis, and reverse phenotyping.

**Results:**

Three unrelated individuals presented in late adolescence for differential diagnosis of incidental microcystic-hyperechogenic kidneys with preserved kidney and liver function. Upon genetic analysis, we identified a homozygous hypomorphic *PKHD1* missense variant causing pseudodominant inheritance in a family, a large monoallelic *PKDH1*-deletion with atypical transmission, and biallelic *PKD1* missense hypomorphs with recessive inheritance.

**Conclusion:**

By this report, we illustrate clinical presentations associated with atypical PKD-gene alterations beyond traditional modes of inheritance. Large monoallelic *PKHD1*-alterations as well as biallelic hypomorphs of both *PKD1* and *PKHD1* may lead to mild CKD in the absence of prominent macrocyst formation and functional liver impairment. The long-term renal prognosis throughout life, however, remains undetermined. Increased detection of atypical inheritance challenges our current thinking of disease ontology not only in PKD but also in Mendelian disorders in general.

## Introduction

Polycystic kidney disease (PKD) is a genetically and clinically heterogeneous condition. In recent years, an increasing number of genetic alterations were associated with PKD (McConnachie et al., 2020). Apart from ongoing detection of new disease-causing alleles in known PKD genes, novel disease genes, such as *GANAB* (Porath et al., 2016), *ALG9* (Besse et al., 2019), *ALG8* (Besse et al., 2017), *DNAJB11* (Cornec-Le Gall et al., 2018a), and *DZIP1L* (Lu et al., 2017), have been identified. By mode of inheritance, PKD is categorized into autosomal dominant (ADPKD, MIM#173900/MIM#613095) and autosomal recessive (ARPKD, MIM#263200). While ADPKD is a quite frequent adult-onset condition due to monoallelic *PKD1* or *PKD2* variation, ARPKD is a rare childhood-onset disorder.

With an estimated prevalence of about 1:1,000, ADPKD is indeed the leading genetic cause of end-stage kidney disease (ESKD) among adults (Cornec-Le Gall et al., 2018b; Lanktree et al., 2018). Patients with protein truncating *PKD1* variants are at higher risk of developing ESKD early in life than patients with non-truncating *PKD1* variants, and *PKD2* carriers express an even milder form of ADPKD often without requirement of renal replacement therapies throughout life (Hwang et al., 2016; Bergmann et al., 2018). However, even in patients with truncating *PKD1* variants, exceptionally mild renal phenotypes can be found similar to patients with *PKD2* disease (Lanktree et al., 2021).

On the contrary, ARPKD affects about 1:20,000 live births and results from biallelic variants in *PKHD1*, encoding the ciliary protein fibrocystin. Although perinatal manifestation is most common, genetic studies in adults demonstrated that ARPKD is also diagnosed later in life with an almost ADPKD-like phenotype (Cornec-Le Gall et al., 2018b; Schönauer et al., 2020). Similar to ADPKD, genotype–phenotype correlations have been established in ARPKD. While biallelic truncating variants are mostly associated with fetal demise (Bergmann et al., 2005; Erger et al., 2017), combinations of truncating and missense variants can be found in children surviving the first year of life, and biallelic missense variants are commonly associated with milder forms of disease compatible with ESKD in adulthood (Bergmann et al., 2018; Schönauer et al., 2020). Nevertheless, exceptional cases with biallelic truncation surviving the perinatal period (Ebner et al., 2017; Burgmaier et al., 2018) and biallelic missense variants linked to severe courses (Furu et al., 2003; Bergmann et al., 2005) were reported. Other studies suggested an impact of the type of affected PKHD1-protein domain on the clinical phenotype (Furu et al., 2003; Bergmann et al., 2005).

Clinical presentations of ADPKD and ARPKD are hence genotype-dependent ranging from fetal demise to mild chronic kidney disease (CKD) in adults (Bergmann et al., 2018). In addition to CKD, hepatic involvement presents differently in ADPKD and ARPKD. While in ADPKD, cystic liver enlargement overlapping with autosomal dominant polycystic liver disease (ADPLD) is a characteristic hallmark, ARPKD often leads to rather microcystic liver fibrosis and more often results in consecutive liver transplantation (Cornec-Le Gall et al., 2018b).

Besides classic ADPKD or ARPKD, other rare cystic kidney disorders include nephronophthisis-related ciliopathies, based on biallelic variants in multiple genes encoding ciliary and centrosomal proteins (Bergmann et al., 2018; Adamiok-Ostrowska and Piekiełko-Witkowska, 2020).

Before the advent of next-generation sequencing (NGS) techniques, establishing the correct diagnosis was mainly based on clinical presentation and kidney imaging. NGS has since increased diagnostic accuracy, also in PKD, and led to an improved understanding of the underlying disease mechanisms (Obeidova et al., 2020).

While exemptions from dominant and recessive inheritance in ADPKD and ARPKD have been reported before (Mantovani et al., 2020; Obeidova et al., 2020), we aim at illustrating the complexity of PKD genetics through peculiar genetic alterations of both *PKD1* and *PKHD1* in three young adult females and one male infant. The presented examples demonstrate unusual modes of inheritance and exceptional clinical presentations, challenging the given disease ontology in PKD.

## Materials and Methods

### Patients

Index patients and their families were recruited from the outpatient clinic for *Hereditary Kidney Disorders* at the University of Leipzig Medical Center. All families were included upon written informed consent approved by the local Institutional Review Board (IRB) at the University of Leipzig, Germany (IRB00001750; #402/16-ek).

### Next-Generation Sequencing and Variant Analysis

All study participants underwent genetic analysis conducted from blood-derived DNA samples by NGS-based gene-panel analysis and multiplex ligation-dependent probe amplification (MLPA). Gene panels included all known PKD-associated genes displayed in Supplementary Table 1 (*PKD1, PKD2*, and *PKHD1*, among others). Further segregation analysis was done by Sanger-sequencing of respective gene variants in available family members. MLPA was used to detect copy number variations (CNVs) and to validate the NGS data set in complex gene regions, ensuring that no structural alteration was overlooked. *In silico* prediction with combined annotation-dependent depletion score (CADD-PHRED v1.6: 23.80) (Rentzsch et al., 2019) was used to categorize newly detected variants in terms of pathogenicity. Variant analysis and interpretation was carried out in accordance with the criteria established by the *American College of Medical Genetics and Genomics* (ACMG) (Richards et al., 2015).

### Reverse Phenotyping

Reverse phenotyping was done in all family members available. Laboratory tests (e.g., serum-creatinine, urea, urine albumin/creatinine ratio) and kidney imaging [renal ultrasound, magnetic resonance imaging (MRI)] were ascertained to derive genotype–phenotype correlations.

## Results

### Family 1: *PKHD1* Disease With Pseudodominant Inheritance

#### Clinical Description

**FIGURE 1:**
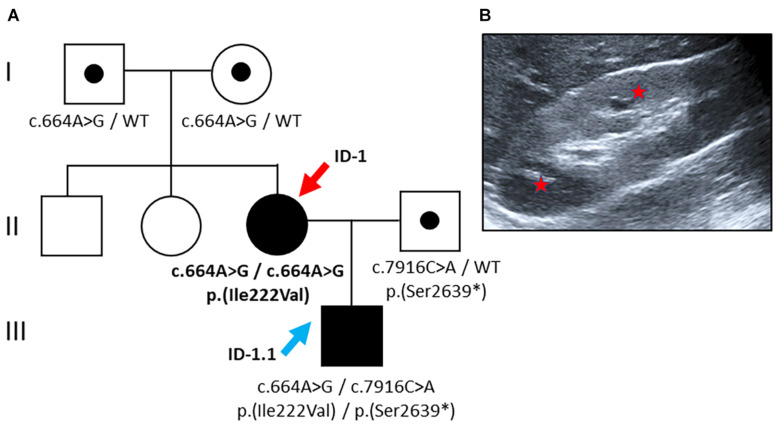
Pedigree and renal imaging (Family 1). **(A)** Family pedigree: Individuals suffering from PKD are indicated as black symbols, and variant carriers are denoted with a black dot. The female index patient (red arrow) carries a homozygous missense variant [NM_138694.3: c.664A > G, p.(Ile222Val)]. Her son (blue arrow) carries compound heterozygous *PKDH1*-variants: maternal missense variant plus a putative paternally transmitted pathogenic nonsense variant [NM_138694.3: c.7916C > A, p.(Ser2639^∗^)]. WT: wild-type. **(B)** Kidney ultrasound of ID-1 at the age of 18 years displaying mild renal phenotype with hyperechogenic parenchyma and microcystic alterations (red stars highlight small cysts).

The asymptomatic index patient (ID 1, female, 18 years) of Family 1 was from non-consanguineous Romany origin without any former medical conditions. She was seeking medical advice during her first pregnancy at 17 years of age. Pregnancy had been complicated by oligohydramnios and fetal hyperechogenic kidneys upon prenatal ultrasound. After birth, the newborn (ID 1.1) demonstrated small cystic alterations of both kidneys in accordance with infantile ARPKD. Extrarenally, bilateral pneumothoraces but no hepatic involvement were diagnosed in the newborn. Upon closer examination, kidney ultrasound in the mother (ID 1) revealed hyperechogenic parenchyma and microcystic alterations at the age of 18 years (Figure 1). Laboratory analysis showed normal serum creatinine with an increased estimated glomerular filtration rate (eGFR CKD-EPI: 122 ml/min/1.73 m^2^), mild proteinuria (albumin/creatinine ratio: 174 mg/g creatinine), and normal liver function (Table 1).

**TABLE 1:** Phenotypic and genotypic characteristics of patients included in this case series.

Patient	FAM 1 – ID 1	FAM 1 – ID 1.1	FAM 2 – ID 2	FAM 3 – ID 3
**Gene**	*PKHD1*	*PKHD1*	*PKHD1*	*PKD1*

**Variant**	hom.c.664A > Gp.(Ile222Val)0.0056%	comp.het. c.664A > Gp.(Ile222Val)0.0056%	het.c.(?_5909)_(12225_?)delEx37_Ex67del	comp. het.c.11723T > Cp.(Leu3908Pro)
1st allele – c. position1st allele – p. positionallele frequency (gnomAD)				
			Novel	Novel
2nd allele – c. position2nd allele – p. positionallele frequency (gnomAD)	c.664A > Gp.(Ile222Val)0.0056%	c.7916C > Ap.(Ser2639*)0.0039%	WT	c.4709C > Tp.(Thr1570Met)0.0031%
Sex	Female	Male	Female	Female
Origin	Romany	Romany	German	German
Parental consanguinity	No	Not reported	No	No
Age at inclusion	18 years	1.5 years	27 years	19 years
Age at 1st manifestation	18 years	Prenatal oligohydramnion	18 years	Prenatal renal hyper-echogenicity
Chronic kidney disease (stage)^#^	G1	G1-2	G1	G1
Proteinuria: albumin/creatinine ratio (mg/g creatinine)	174	31	6	19.5
Increased echogenicity	+	+	+	+
Renal cysts	+Bilateral small cysts	++bilateral multiple cysts	+++bilateral multiple cysts, enlarged kidneys	+++bilateral multiple, tiny cysts “Salt and pepper pattern”
Kidney stones	–	–	+	–
Extrarenal manifestation	–	Bilateral pneumothoraces	–	–
Arterial hypertension	–	+	–	+
Liver phenotype	–	–	–	–

*The variants reported refer to RefSeq NM_138694.3 (*PKDH1*), NM_001009944.2 (*PKD1*).*

*Abbreviations: comp. het., compound heterozygous; hom., homozygous; het, heterozygous; WT, wild-type; y, years; ^#^Chronic kidney disease refers to the classification of the “Kidney Disease: Improving Global Outcomes” (KDIGO) initiative. gnomAD v2.1.1 – population specific allele frequency (http://gnomad.broadinstitute.org/).*

#### Molecular Genetics

With affected individuals in two subsequent generations, the pedigree suggested dominant inheritance (Figure 1). Genetic analyses of the index patient (ID 1), however, revealed a known pathogenic *PKHD1* missense variant [NM_138694.3: c.664A > G, p.(Ile222Val)] in the homozygous state. This variant affects an evolutionary conserved amino acid residue located near the N-terminal extracellular tail of fibrocystin within the second IPT/TIG (Ig-like, plexins, and transcription factors) domain^1^ (Gunay-Aygun et al., 2010). Allele frequency in Europeans (non-Finish) amounts to 0.0056% with no homozygous cases reported in population databases (gnomAD). *In silico* prediction using CADD yielded a score of 16.14 (CADD-PHRED v1.6) (Rentzsch et al., 2019), compatible with pathogenicity in a recessive disease model.

Surprisingly, the newborn, who was diagnosed with congenital kidney disease *in utero*, was found to carry compound heterozygous *PKDH1* variants: the aforementioned maternal missense variant [c.664A > G, p.(Ile222Val)] in exon 9 plus another pathogenic *PKHD1* nonsense variant in exon 50 out of 67 [NM_138694.3: c.7916C > A, p.(Ser2639^∗^)] on the paternal allele (CADD-PHRED v1.6: 40.00) (Denamur et al., 2010; Rentzsch et al., 2019). For the latter variant, population allele frequency is estimated at 0.0039% (gnomAD). Its deduced impact on the protein level leads to the introduction of a stop codon after the sixth PbH1 (parallel beta-helix) repeat resulting in the loss of the critical second G8 domain containing eight conserved glycine residues likely important for protein stability (see text foot note 1) (He et al., 2006).

### Family 2: *PKHD1* Disease With Atypical Transmission of Monoallelic CNV

#### Clinical Description

**FIGURE 2:**
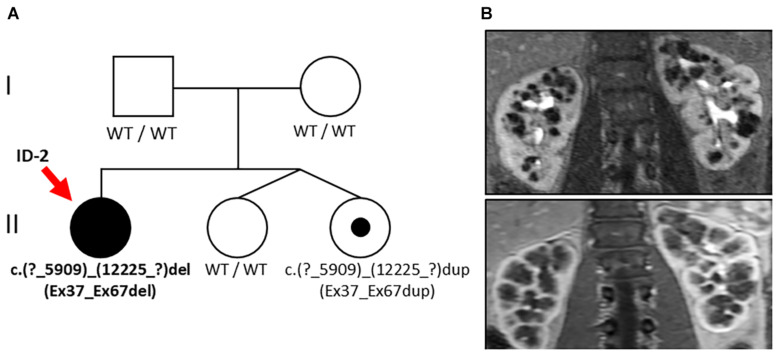
Pedigree and renal imaging (Family 2). **(A)** Family pedigree: Individuals suffering from PKD are indicated as black symbols and variant carriers with a black dot. Genetic analysis in the index patient (ID-2, red arrow) revealed the large heterozygous deletion NM_138694.3: c.(?_5909)_(12225_?)del of *PKHD1* (Ex37_Ex67del) and familial segregation analysis in one younger sibling yielded duplication of the same alteration c.(?_5909)_(12225_?)dup (Ex37_Ex67dup). **(B)** Abdominal MRI of ID 2 at the age of 18 years showing bilateral cystic kidney enlargement.

In family 2 from central Europe, the index patient (ID 2; female, 27 years) came to obtain medical advice after previous genetic testing. At the age of 18 years, ID 2 was hospitalized due to repeated urinary tract infections and conspicuous renal morphology with increased parenchymal echogenicity. Abdominal MRI showed symmetric enlargement of both kidneys [diameter (*length* × *width*): right: 130 × 60 mm; left: 146 × 72 mm] with small cystic alterations but regular hepatic morphology (Figure 2). At the time of presentation in our clinic, the kidney function was preserved (eGFR CKD-EPI: 130 ml/min/1.73 m^2^), and no proteinuria was detected (albumin/creatinine ratio: 6 mg/g) (Table 1). The siblings and parents of the patient showed no renal or liver phenotype.

#### Molecular Genetics

Genetic analysis revealed a large heterozygous deletion of *PKHD1* c.(?_5909)_(12225_?)del (designated as Ex37_Ex67del) encompassing the extracellular domains of PbH1-9 and one of the critical G8 repeats of fibrocystin close to the cell membrane (see text foot note 1). Familial segregation analysis of both parents showed no genetic alteration of *PKHD1*, suggesting the *de novo* nature of this finding. However, further segregation analysis including the younger siblings of ID 2 who were non-identical twins yielded duplication of the same region c.(?_5909)_(12225_?)dup (designated as Ex37_Ex67dup) in one of the twin sisters without clinical manifestation.

### Family 3: *PKD1* Disease With Recessive Inheritance

#### Clinical Description

**FIGURE 3:**
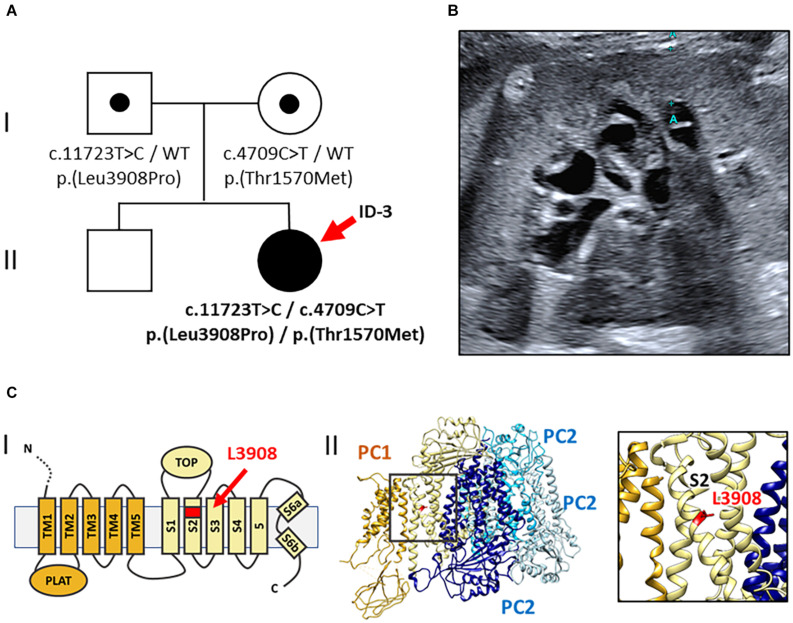
Pedigree, renal imaging, and 3D-PC1-protein structure (Family 3). **(A)** Family pedigree: ID-3 (red arrow) with compound heterozygous rare *PKD1* missense variants both of uncertain significance [NM_001009944.2: c.11723T > C, p.(Leu3908Pro) and c.4709C > T, p.(Thr1570Met)]. WT: wild-type. **(B)** Kidney ultrasound of ID-3 at the age of 19 years shows enlarged, microcystic-hyperechogenic kidneys. **(C)** Localization of the novel variant p.(Leu3908Pro) in the PC1 protein structure. **(I)** Schematic illustration of the PC1 transmembrane region; L3908 is located in transmembrane helix S2 (red arrow). **(II)** Cryo-EM structure of human PC1/PC2 complex (pdb 6A70) (left) and zoom-in view (right) showing the position of L3908 in helix S2 (red) (Su et al., 2018).

This index patient (ID 3, female, 19 years) of central European descent had been congenitally diagnosed with infantile PKD and suspected ARPKD because of early-onset cystic kidneys. The kidney function, however, was completely preserved (eGFR CKD-EPI 133 ml/min/1.73 m^2^) with merely mild proteinuria (albumin/creatinine ratio: 29 mg/g creatinine) (Table 1). While liver imaging presented normal, kidney ultrasound showed bilaterally enlarged kidneys [diameter (*length* × *width*): right: 117 × 52 mm; left: 120 × 57 mm] with increased parenchymal echogenicity and abnormal corticomedullary differentiation (the so-called “salt and pepper” pattern) (Iorio et al., 2020) (Figure 3).

#### Molecular Genetics

Gene-panel analysis of PKD-associated genes in the index patient yielded two rare heterozygous *PKD1* missense variants. Diagnostic variants in *PKHD1* or other known kidney cyst genes were not detected. Both *PKD1* variants were of uncertain significance by ACMG classification [NM_001009944.2: c.11723T > C, p.(Leu3908Pro) and NM_001009944.2: c.4709C > T, p.(Thr1570Met)] (Kane et al., 2006; Richards et al., 2015). Parental segregation analysis confirmed compound heterozygosity in the index patient with c.11723T > C [p.(Leu3908Pro)] being paternally inherited and c.4709C > T [p.(Thr1570Met)] being maternally transmitted. Of note, clinical examination of both parents was unremarkable, including absence of kidney and liver cysts at the age of 32 years. The first *PKD1* variant is novel, neither reported in population databases (gnomAD) nor disease databases [HGMD Professional Version 2020.4/Mayo PKD-database^2^ (Stenson et al., 2020)]. The respective amino acid residue Leu3908 is evolutionary conserved in vertebrates belonging to the transmembrane helix S2 domain of polycystin 1 (Figure 3)(Su et al., 2018). Introduction of a proline residue into the alpha-helix is likely deleterious, leading to conformational impairments. Hence, *in silico* prediction using CADD also suggested pathogenicity (CADD-PHRED v1.6: 23.80)(Rentzsch et al., 2019).

The second *PKD1* variant [c.4709C > T, p.(Thr1570Met)] was previously reported in the literature (Vujic et al., 2010), where it was found to be associated with *in utero* onset disease in the homozygous state but incomplete penetrance in heterozygous carriers. Thr1570 constitutes a highly conserved residue in vertebrates and localizes to the PKD11 domain (CADD-PHRED v1.6: 26.30)(Rentzsch et al., 2019). Its allele frequency in Europeans is 0.0031% without homozygous findings in population databases (gnomAD).

## Discussion

Genetic variant interpretation and its clinical correlation has become one of the major challenges of our times. Clinical geneticists and treating physicians are increasingly confronted with new rare variants that require meaningful translation into clinical practice. Furthermore, genetic results often question our traditional categories and force us to take a new perspective on disease classification and terminology. This dilemma is exemplified by the presented cases of three young females with mild cystic kidney alterations illustrating the difficulties and chances of adequate genetic counseling and disease terminology in the era of broadly accessible genetic testing. All index patients presented clinically asymptomatic with preserved kidney function as young adults.

The common denominator is hampered disease prediction through atypical and novel genetic findings with little clinical experience derivable from previously published cases. When it comes to disease ontology, neither single heterozygous *PKHD1* germline variants nor biallelic *PKD1* hypomorphs do match the given terminology of autosomal-recessive and autosomal-dominant PKD but mimic each other’s renal manifestation. Therefore, common risk stratification tools like the Mayo imaging classification (Irazabal et al., 2015) or the PROPKD-score (Cornec-Le Gall et al., 2016) are not applicable, complicating genetic counseling and decision-making in respective families.

The pedigree of family 1 revealed affected family members in two subsequent generations suggesting dominant inheritance. Genetic testing, however, showed *PKHD1* variants defining ARPKD and thereby led to correction of previous assumptions. While pseudodominant inheritance was reported in other Mendelian kidney diseases, e.g., Gitelman’s disease (de La Faille et al., 2011), this is the first report of pseudodominant inheritance in ARPKD. The two affected family members, the index patient (Fam 1 – ID 1) and her newborn son (ID 1.1), impressively illustrate the broad clinical spectrum of ARPKD ranging from severe congenital manifestation to mild CKD in adults depending on the nature and combination of respective *PKHD1* alleles (Burgmaier et al., 2019). The homozygous pathogenic *PKHD1* missense variant [p.(Ile222Val)] in the index patient leads to a mild course of disease potentially even compatible with normal life expectancy. The very missense variant has been reported frequently in compound heterozygous state in patients with ARPKD before (Bergmann et al., 2005; Denamur et al., 2010; Gunay-Aygun et al., 2010; Brinkert et al., 2013; Obeidova et al., 2020), but has not yet been reported homozygously to the best of our knowledge. Therefore, long-term prognoses remain speculative. Asymptomatic presentation with normal renal and liver function at age 18 suggests that biallelic occurrence of the Ile222Val missense variant is linked to a mild renal phenotype and that only additional second/third hits result in more severe courses of ARPKD. This theory is supported by the clinical presentation of her newborn carrying an additional known *PKHD1* null allele (p.Ser2639^∗^), which is obviously associated with aggravated disease severity (Denamur et al., 2010; Melchionda et al., 2016; Szabó et al., 2018).

The second family represents another example of mild ARPKD in adults. This time, the index patient (Fam 2 – ID 2) was found to carry a large heterozygous deletion in *PKHD1* (Ex37_Ex67del). As clinically healthy parents showed *PKHD1* wild-type sequences, *de novo* nature of the deletion was assumed until further segregation analysis in the sister yielded duplication of the very same region without clinical manifestation. This unexpected finding leads to verification of our initial assumption and established yet another mechanism of inheritance in terms of recurrent unequal meiotic crossovers or an unbalanced cryptic chromosomal translocation. This case demonstrates sporadic manifestation of ARPKD based on single heterozygous alterations of *PKHD1*, an observation that was increasingly reported recently (Besse et al., 2017). Additionally, data in mice suggest that heterozygous *PKHD1* carriers may develop ARPKD-associated liver cysts only later in life after the occurrence of a second hit affecting the *PKHD1* wild-type allele in somatic tissues (Shan et al., 2019; Besse et al., 2020). However, given the large size and complex exon structure of *PKHD1*, we cannot exclude a deep intronic splice variant *in trans* with the herein described deletion as an alternative explanation (Michel-Calemard et al., 2009; Chen et al., 2019). *PKHD1* is not sufficiently expressed in blood to be easily accessible to RNA sequencing approaches, but future analyses from cultured urinary renal epithelial cells might enable screening for these rare variants in atypical cases with single *PKHD1* variants.

Lastly, the third case is an example for biallelic hypomorphic *PKD1* variants mimicking ARPKD. First, the index patient presented in childhood with enlarged kidneys and morphological signs of ARPKD upon kidney ultrasound (Iorio et al., 2020); second, the pedigree of the family indicated recessive inheritance. Surprisingly, genetic analysis in the index patient yielded no diagnostic findings in *PKHD1*, but compound heterozygosity for two rare *PKD1* missense variants, both of uncertain significance. One of them [c.4709C > T, p.(Thr1570Met)], however, was previously associated with early-onset ADPKD in the homozygous state (Vujic et al., 2010). In this case, two *PKD1* variants *in trans* manifested as phenocopy of ARPKD. One of these hypomorphic alleles alone likely results in a normal phenotype, but two alleles *in trans* cause early-onset cystic kidney disease. As cyst initiation is assumed to depend on the dosage of functional Polycystin 1 (Rossetti et al., 2009), the combination of two hypomorphic variants could additively reduce the *PKD1* function below a critical threshold to result in clinically apparent disease. Coincidence of biallelic hypomorphic *PKD1* variants represents an example for PKD without an apparent family history (Iliuta et al., 2017).

Given these examples, we suggest revisiting and adapting the classification of genetic PKD to enable the description of the apparent continuum of AR/ADPKD genotypes and phenotypes. In a subset of cases, clinical presentation and associated genetic findings do not fit into the current ontology. As a first step, a more flexible disease terminology including the respective gene name was suggested in 2018 without being widely adopted to date (Cornec-Le Gall et al., 2018b). In other renal conditions, however, such as autosomal-dominant tubulointerstitial kidney disease (ADTKD), a new disease classification was successfully established, combining the mode of inheritance, major clinical manifestation, and underlying genetic defect in one term (ADTKD-*UMOD*; ADTKD-*REN*, ADTKD-*MUC1*, and ADTKD-*HNF1B*) (Bleyer et al., 2017).

In summary, this report demonstrates the genetic complexity of PKD. Attributing the correct diagnosis and providing adequate genetic counseling to affected patients and their families is not self-evident. Atypical presentations should prompt immediate genetic testing to increase diagnostic accuracy by verification or correction of clinically assumed diagnoses.

## Data Availability Statement

The data analyzed in this study is subject to the following licenses/restrictions: requests to access these datasets should be directed to JH, jan.halbritter@medizin.uni-leipzig.de.

## Ethics Statement

The studies involving human participants were reviewed and approved by local Institutional Review Board (IRB) at the Universities of Leipzig, Germany (IRB00001750; #402/16-ek). Written informed consent to participate in this study was provided by the participants’ legal guardian/next of kin.

## Author Contributions

JF and JH contributed to the conception and design of the study, and helped in investigation. JF wrote the first draft of the manuscript. RS, JM, and BP wrote the sections of the manuscript. JF, RS, MN, BP, and JH contributed to the analysis. JF and RS helped in visualization. All authors contributed to manuscript revision, read, and approved the submitted version.

## Conflict of Interest

The authors declare that the research was conducted in the absence of any commercial or financial relationships that could be construed as a potential conflict of interest.

## Footnotes

1 https://www.uniprot.org/uniprot/P08F94

2 http://pkdb.pkdcure.org
