# Supplementary material for: Challenging Disease Ontology by Instances of Atypical PKHD1 and PKD1 Genetics
Source: Front Genet. 2021 Jun 25;12:682565. doi: 10.3389/fgene.2021.682565 (PMC8267867; doi:10.3389/fgene.2021.682565)
Supplement: Supplementary file 1 [file Table_1.DOCX]

**Supplement 1: Panel diagnostic of cystic genes:**

*CEP290
INVS
IQCB1
NPHP1
NPHP3
NPHP4
TMEM67
AGXT
ANKS6
CEP164
CEP83
DCDC2
FAN1
GLIS2
IFT172
MAP7D3
MAPKBP1
NEK8
RPGRIP1L
SDCCAG8
SLC41A1
TRAF3IP1
TTC21B
WDR19
XPNPEP3
ZNF423
ACE
ALG9
ANKS6
BICC1
DICER1
DNAJB11
DZIP1L
GANAB
HNF1B
INVS
LRP5
NPHP3
OFD1
PAX2
PKD1
PKD2
PKHD1
PMM2
TMEM67
UMOD*
